# Twin epidemics of new and prevalent hepatitis C infections in Canada: BC Hepatitis Testers Cohort

**DOI:** 10.1186/s12879-016-1683-z

**Published:** 2016-07-19

**Authors:** Naveed Zafar Janjua, Amanda Yu, Margot Kuo, Maria Alvarez, Darrel Cook, Jason Wong, Mark W. Tyndall, Mel Krajden

**Affiliations:** Clinical Prevention Services, British Columbia Centre for Disease Control, Vancouver, BC Canada; School of Population and Public Health, University of British Columbia, Vancouver, BC Canada; Department of Pathology and Laboratory Medicine, University of British Columbia, Vancouver, BC Canada

**Keywords:** HCV, Hepatitis B, HIV, Epidemiology, Screening, Cohort study

## Abstract

**Background:**

We characterized the twin epidemics of new and prevalent hepatitis C virus (HCV) infections in British Columbia, Canada to inform prevention, care and treatment programs.

**Methods:**

The BC Hepatitis Testers Cohort (BC-HTC) includes individuals tested for HCV, HIV or reported as a case of HBV, HCV, HIV or active TB between 1990–2013 linked with data on their medical visits, hospitalizations, cancers, prescription drugs and mortality. Prevalent infection was defined as being anti-HCV positive at first test. Those with a negative test followed by a positive test were considered seroconverters or new infections.

**Results:**

Of 1,132,855 individuals tested for HCV, 64,634 (5.8 %) were positive and an additional 3092 cases tested positive elsewhere for a total of 67,726. Of 55,781 HCV positive individuals alive at the end of 2013, 7064 were seroconverters while 48,717 had prevalent infection at diagnosis. The HCV positivity rate (11.2 %) was highest in birth cohort 1945–1964 which declined over time. New infections were more likely to be male, 15–34 years of age (born 1965-1984), HIV- or HBV-coinfected, socioeconomically disadvantaged, have problematic drug and alcohol use and a mental health illness. The profile was similar for individuals with prevalent infection, except for lower odds of HBV-coinfection, major mental health diagnoses and birth cohort >1975.

**Conclusions:**

The HCV positivity rate is highest in birth cohort 1945–1964 which represents most prevalent infections. New infections occur in younger birth cohorts who are commonly coinfected with HIV and/or HBV, socioeconomically marginalized, and living with mental illness and addictions.

**Electronic supplementary material:**

The online version of this article (doi:10.1186/s12879-016-1683-z) contains supplementary material, which is available to authorized users.

## Background

Hepatitis C virus (HCV) is a major global public health problem. Worldwide, approximately 184 million people are infected [1], and in Canada, 210,753–461,517 (0.66 %-1.3 %) people are estimated to be infected with HCV [2]. In British Columbia (BC), the annual rate of newly identified HCV cases is about 50 % higher than the national average [3]. Untreated HCV infected individuals have a ~5-fold increased all-cause and ~20-fold increased risk of liver-related mortality [4]. While treatment reduces both morbidity and mortality, only about 10 % of infected individuals have been treated in BC [5]. Newer well-tolerated and highly effective direct acting antiviral agents, with cure rate approaching 95 %, are expected to provide new opportunities to prevent progressive liver disease [6, 7]. However, a large number of people are unaware of their infection [2, 8]. Characterising individuals who have been tested and who test positive can help identify prevention, testing, care and treatment strategies and programs to reach people most in need.

In most developed countries, the different HCV affected populations can be defined from their risk characteristics that can inform prevention, testing and treatment policies. HCV affected individuals could be classified into two broad groups: prevalent and new infections. Prevalent HCV infections represent infections acquired in the distant past and these individuals are usually not involved in ongoing risk activities. New infections represent ongoing transmission usually occurring in people with high risk activities such as injection drug use. These two groups differ not only in terms of demographics and risk factors but also have varying levels of onward transmission risk and hence unique needs for engagement in prevention and care. Characterization of these population groups using testing patterns, co-occurrence of other infections (HIV, tuberculosis [TB], sexually transmitted infections [STIs], hepatitis B [HBV]), and social risk factors (addiction and mental illness) could inform the design of prevention, testing and treatment programs tailored to the specific needs of these groups. In this paper, we characterize twin epidemics of HCV infection by describing characteristics and associated factors of acute and prevalent infections using data from the BC Hepatitis Testers Cohort (BC-HTC). We further characterize the HCV disease burden by birth cohorts and present positivity by birth cohort to inform birth cohort screening.

## Methods

### The cohort and study population

The BC Hepatitis Testers Cohort(BC-HTC) includes all individuals tested for HCV or HIV at the BC Public Health Laboratory or reported to public health as a confirmed case of HCV, HBV, HIV/AIDS or active TB. The cohort is linked with medical visits, hospitalizations, prescription drugs, cancers and deaths [9–13] (Table 1). Details of the cohort creation, linkage and characteristics of matched and unmatched individuals have been reported previously [14]. The overall linkage rate for HCV was >85 % and approached 90 % after 2006.

**Table 1:** Criteria and Data Sources for the BC Hepatitis Testers Cohort (BC-HTC) and current analysis

Criteria for Inclusion in BC-HTC All individuals: • tested at the centralized provincial laboratory for HCV or HIV OR • reported in BC as a confirmed case of HCV OR • reported in BC enhanced surveillance system as a confirmed case of HIV or AIDS (all reports) OR • reported in BC as a confirmed case of HBV OR • reported in BC as a confirmed case of active TB (latent cases excluded) OR • included in BC Enhanced Strain Surveillance System (EHSSS) as an acute HBV or HCV case	
All individuals meeting at least one the above criteria were linked internally across all their tests and case reports. Those with a valid personal health number (PHN) were then sent for deterministic linkage with province-wide Cancer and Ministry of Health (MoH) datasets	
Provincial Communicable Disease Data Sources • BC-PHL HIV laboratory testing datasets (tests: ELISA, Western blot, NAAT, p24, culture) • BC-PHL HCV laboratory tests datasets (tests: antibody, HCV RNA, genotyping) • HIV/AIDS Information System (HAISYS) (public health HIV/AIDS case reports) • Integrated Public Health information System (iPHIS) (public health case reports of HCV, HBV, and TB) • Enhanced Strain Surveillance System (EHSSS) (risk factor data on a subset of acute HCV and acute HBV cases)	Data Date Ranges: HIV lab data: 1988–2013 HCV lab data: 1992–2013 HAISYS: 1980–2013 iPHIS: 1990–2013 EHSSS: 2000–2013
Cancer and MoH Administrative Data Sources • BC Cancer Registry (BCCR) (primary tumour registry, excludes metastatic cancers) • Discharge Abstracts Dataset (DAD) (hospitalization records) • Medical Services Plan (MSP) (physician diagnostic and billing data) • PharmaCare/PharmaNet (Pharma) (prescription drug dispensations) • BC Vital Statistics (VS) (deaths registry)	Data Date Ranges: BCCR: 1970–2012 DAD: 1985–2013Q1 MSP: 1990–2012 Pharma: 1985–2012 VS: 1985–2013
The final BC-HTC comprises all individuals successfully linked on PHN to the MoH Client Roster (a registry of all BC residents enrolled in the publicly-funded universal healthcare system)	
Eligibility for current analysis • all subjects included in final, linked BC-HTC with at least one valid HCV test (antibody, HCV RNA, or genotype) (1992–2013) or HCV case report (1990–2013) • subjects were censored for analysis date of death or December 31, 2013, whichever came first • excluded all data preceding subject’s recorded date of birth • excluded all data occurring more than 7 days after subject’s recorded date of death • excluded HCV antibody tests in infants less than 18 months in the absence of HCV RNA confirmatory testing	

BC-HTC participants with at least one HCV test (antibody, HCV RNA, or genotype) or HCV case report contributed to the analysis from their first HCV test date (beginning 1992) or report (beginning 1990) through date of death or December 31, 2013, whichever was earliest (Table 1). We excluded data which preceded a participants’ birth date or occurred >7 days after the death date. Antibody tests in infants <18 months in the absence of HCV RNA testing were excluded as passive maternal antibody could not be ruled out.

### Definitions

A HCV case was defined as an individual testing positive for either HCV antibody or RNA or genotype or was reported as a HCV case through the public health reporting system [15]. We considered participants who were HCV antibody positive at their first test on record as having prevalent infection as, without a prior negative test, a seroconversion interval cannot be established and, based on natural history, most will be chronically infected. Those cases not found in laboratory data but reported by public health as confirmed HCV were considered prevalent infections. Individuals with a negative followed by a positive test were considered seroconverters or new infections. Individuals who seroconverted within 24 months of the last negative test were termed 24-month seroconverters, while those who seroconverted after 24 months are >24-months seroconverters. For surveillance purposes in BC, acute HCV infection is defined as seroconversion within the previous 12 months [15]. Since the characteristics of 12- and 24-month seroconverters were similar as presented in Additional file 1: Table S2, we report 0–24-month seroconverters as acute HCV cases. Co-infection was defined as being infected with HIV, HBV or active TB together with HCV.

Socioeconomic status was assessed using the Québec Index of Material and Social Deprivation, a widely used index based on Canadian Census data [16]. Data from each census, conducted every 5 years, was applied to two years before and two years after the census year. For the 2011 census, the index is based on the data product CensusPlus to address non-response related bias in the 2011 National Household Survey [17].

Assessment of illicit drug use, mental illnesses, and problematic alcohol use was based on having diagnostic codes in administrative health datasets evaluated across all data prior to and within 3 years of the first positive test or last negative test (Additional file 1: Table S1).

### Statistical analysis

We compared the characteristics of HCV positive cases (24-month seroconverters, >24-month seroconverters and prevalent cases) and negative groups using the chi-square test for categorical factors or the Wilcoxon-Mann-Whitney test for continuous variables. We calculated HCV positivity across birth cohorts and compared their characteristics. Positivity rate is defined as number of hepatitis C diagnoses divided by the total number of people tested for hepatitis C. We performed multinomial logistic regression to assess the factors associated with new and prevalent infections at diagnosis compared to HCV negatives.

## Results

### Participant Profile, testing and infection prevalence

The cohort included 1,132,855 subjects tested for hepatitis C from 1992-2013. Of these, 64,634 (5.7 %) were HCV positive. An additional 3092 cases tested elsewhere and reported to the public health surveillance system from 1990-2013 resulted in a total of 67,726 HCV cases. Of HCV positive individuals, 11,945 (17.6 %) have died and 55,781 were alive at the end of 2013. Among 1,068,221 HCV negative individuals, 79,840 (7.5 %) have died and 988,381 were alive at the end of 2013. The following results are from individuals alive as of December 31, 2013 unless specified otherwise.

### Characteristics of HCV positive and negative individuals

For HCV positive individuals, the median age at their first test was 40 years and 35 years for HCV negative individuals. A comparatively small proportion (12.2 %) of HCV positive individuals were born after 1975 compared to those HCV negative (37.6 %; *p* < 0.001). A higher proportion of HCV positives compared to negatives were male (64.4 % vs. 44.1 %; *p* < 0.001). Infection with HIV (5.7 % vs. 0.5 %; *p* < 0.001), HBV (3.7 % vs. 2.0 %; *p* < 0.001) and active TB (0.5 % vs 0.2 %; *p* < 0.001) were significantly higher in HCV positives than negatives. There was a graded increase in percent HCV positivite with increases in material (most privileged: 3.2 % vs. most deprived: 8.0 %) and social deprivation (most privileged: 3.1 % vs. most deprived: 7.7 %). There were fewer privileged (material: 13.4 % vs. 22.0 %/social: 11.0 % vs 18.5 %) and more deprived (material: 29.6 % vs. 18.8 %; *p* < 0.001/social: 37.7 % vs. 24.8 %; *p* < 0.001) individuals among HCV positives compared to HCV negatives. The proportions with illicit drug use (35.2 % vs. 7.0 %), problematic alcohol use (21.7 % vs. 6.2 %) and mental health conditions (16.0 % vs. 12.0 %) at diagnosis were also higher among HCV positives compared to HCV negatives (Table 2).

**Table 2:** Characteristics of HCV positive and negative individuals, BC-HTC, Canada, 1990-2013

	HCV + ve group				HCV -ve group
	M24 Sero^a^	>M24 Sero^b^	Prevalent HCV	All Positives	All Negatives
	*N* = 3628	*N* = 3436	*N* = 48717	*N* = 55781	*N* = 988381
	N (%)	N (%)	N (%)	N (%)	N (%)
Age at baseline^c^					
< 15	17 (0.5)	1 (0.0)	420 (0.9)	438 (0.8)	20289 (2.1)
15-24	962 (26.5)	410 (11.9)	2410 (5.0)	3782 (6.8)	145326 (14.7)
25-34	1477 (40.7)	1234 (35.9)	9907 (20.3)	12618 (22.6)	259347 (26.2)
35-44	827 (22.8)	1106 (32.2)	16534 (33.9)	18467 (33.1)	220626 (22.3)
45-54	275 (7.6)	522 (15.2)	13068 (26.8)	13865 (24.9)	163134 (16.5)
≥ 55	70 (1.9)	163 (4.7)	6378 (13.1)	6611 (11.9)	179659 (18.2)
Median [IQR]	30 [24-37]	35 [28-43]	42 [34-49]	41 [33-48]	37 [28-50]
Age at 1st test					
< 15	100 (2.8)	84 (2.4)	421 (0.9)	605 (1.1)	25860 (2.6)
15-24	1485 (40.9)	1128 (32.8)	2422 (5.0)	5035 (9.0)	189747 (19.2)
25-34	1225 (33.8)	1278 (37.2)	9927 (20.4)	12430 (22.3)	261056 (26.4)
35-44	605 (16.7)	712 (20.7)	16590 (34.1)	17907 (32.1)	208398 (21.1)
45-54	169 (4.7)	195 (5.7)	13063 (26.8)	13427 (24.1)	147688 (14.9)
≥ 55	44 (1.2)	39 (1.1)	6294 (12.9)	6377 (11.4)	155632 (15.8)
Median [IQR]	26 [20-34]	28 [22-35]	42 [34-49]	40 [32-48]	35 [26-48]
Birth year					
< 1945	31 (0.9)	35 (1.0)	3102 (6.4)	3168 (5.7)	95848 (9.7)
1945-1964	776 (21.4)	1037 (30.2)	32133 (66.0)	33946 (60.9)	310845 (31.5)
1965-1974	1176 (32.4)	1214 (35.3)	9493 (19.5)	11883 (21.3)	209956 (21.2)
≥ 1975	1645 (45.3)	1150 (33.5)	3989 (8.2)	6784 (12.2)	371732 (37.6)
Sex					
Female	1728 (47.6)	1380 (40.2)	16741 (34.4)	19849 (35.6)	551952 (55.8)
Male	1900 (52.4)	2056 (59.8)	31970 (65.6)	35926 (64.4)	436287 (44.1)
Unknown	0 (0.0)	0 (0.0)	6 (0.0)	6 (0.0)	142 (0.0)
Year of diagnosis					
1990-1994	85 (2.3)	4 (0.1)	3978 (8.2)	4067 (7.3)	3913 (0.4)
1995-1999	726 (20.0)	353 (10.3)	17936 (36.8)	19015 (34.1)	104863 (10.6)
2000-2004	1177 (32.4)	939 (27.3)	11733 (24.1)	13849 (24.8)	172888 (17.5)
2005-2009	994 (27.4)	1280 (37.3)	9130 (18.7)	11404 (20.4)	272886 (27.6)
2010-2013	646 (17.8)	860 (25.0)	5940 (12.2)	7446 (13.4)	433831 (43.9)
HIV/AIDS at baseline					
Unknown	3450 (95.1)	3207 (93.3)	47568 (97.6)	54225 (97.2)	984182 (99.6)
Yes	178 (4.9)	229 (6.7)	1149 (2.4)	1556 (2.8)	4199 (0.4)
HIV/AIDS (ever)					
Unknown	3264 (90.0)	3104 (90.3)	46257 (95.0)	52625 (94.3)	983694 (99.5)
Yes	364 (10.0)	332 (9.7)	2460 (5.0)	3156 (5.7)	4687 (0.5)
HBV at baseline					
No/Unknown	3528 (97.2)	3349 (97.5)	48197 (98.9)	55074 (98.7)	974223 (98.6)
Yes	100 (2.8)	87 (2.5)	520 (1.1)	707 (1.3)	14158 (1.4)
HBV (ever)					
No/Unknown	3453 (95.2)	3326 (96.8)	46927 (96.3)	53706 (96.3)	968459 (98.0)
Yes	175 (4.8)	110 (3.2)	1790 (3.7)	2075 (3.7)	19922 (2.0)
Active TB at baseline					
No/Unknown	3621 (99.8)	3428 (99.8)	48634 (99.8)	55683 (99.8)	986909 (99.9)
Yes	7 (0.2)	8 (0.2)	83 (0.2)	98 (0.2)	1472 (0.1)
Active TB (ever)					
No/Unknown	3611 (99.5)	3415 (99.4)	48466 (99.5)	55492 (99.5)	986473 (99.8)
Yes	17 (0.5)	21 (0.6)	251 (0.5)	289 (0.5)	1908 (0.2)
Material deprivation quintile at baseline					
Unknown	96 (2.7)	79 (2.3)	2316 (4.8)	2491 (4.5)	14773 (1.5)
Q1 (most privileged)	452 (12.5)	444 (12.9)	6247 (12.8)	7143 (12.8)	214007 (21.7)
Q2	526 (14.5)	463 (13.5)	7500 (15.4)	8489 (15.2)	187587 (19)
Q3	531 (14.6)	585 (17.0)	8522 (17.5)	9638 (17.3)	192265 (19.5)
Q4	876 (24.2)	774 (22.5)	10580 (21.7)	12230 (21.9)	197116 (19.9)
Q5 (most deprived)	1147 (31.6)	1091 (31.8)	13552 (27.8)	15790 (28.3)	182633 (18.5)
Social deprivation quintile at baseline					
Unknown	96 (2.7)	79 (2.3)	2316 (4.8)	2491 (4.5)	14773 (1.5)
Q1 (most privileged)	271 (7.5)	283 (8.2)	5302 (10.9)	5856 (10.5)	180214 (18.2)
Q2	349 (9.6)	337 (9.8)	6194 (12.7)	6880 (12.3)	175098 (17.7)
Q3	540 (14.9)	463 (13.5)	8094 (16.6)	9097 (16.3)	175715 (17.8)
Q4	703 (19.4)	720 (21.0)	9972 (20.5)	11395 (20.4)	201551 (20.4)
Q5 (most deprived)	1669 (46.0)	1554 (45.2)	16839 (34.6)	20062 (36)	241030 (24.4)
Mental health at baseline^d^					
No/Unknown	2199 (64.5)	2096 (65.0)	40934 (86.7)	45229 (84.0)	729398 (88.0)
Yes	1209 (35.5)	1130 (35.0)	6297 (13.3)	8636 (16.0)	99054 (12.0)
Mental health 3yrs pre-baseline^d^					
No/Unknown	2590 (76.0)	2653 (82.2)	43518 (92.1)	48761 (90.5)	781042 (94.3)
Yes	818 (24.0)	573 (17.8)	3713 (7.9)	5104 (9.5)	47410 (5.7)
Illicit drug use at baseline^d^					
No/Unknown	910 (26.7)	942 (29.2)	33036 (69.9)	34888 (64.8)	770499 (93.0)
Yes	2498 (73.3)	2284 (70.8)	14195 (30.1)	18977 (35.2)	57953 (7.0)
Illicit drug use 3yrs pre-baseline^d^					
No/Unknown	1084 (31.8)	1433 (44.4)	36193 (76.6)	38710 (71.9)	795325 (96.0)
Yes	2324 (68.2)	1793 (55.6)	11038 (23.4)	15155 (28.1)	33127 (4.0)
Problematic alcohol use at baseline^d^					
No/Unknown	2083 (61.1)	1896 (58.8)	38178 (80.8)	42157 (78.3)	777271 (93.8)
Yes	1325 (38.9)	1330 (41.2)	9053 (19.2)	11708 (21.7)	51181 (6.2)
Problematic alcohol use 3yrs pre-baseline^d^					
No/Unknown	2521 (74.0)	2555 (79.2)	41709 (88.3)	46785 (86.9)	802492 (96.9)
Yes	887 (26.0)	671 (20.8)	5522 (11.7)	7080 (13.1)	25960 (3.1)

^a^24-month Sero: Individuals who seroconverted within 24 months of the last negative test

^b^Individuals who seroconverted after 24 months of the last negative test

^c^Baseline is defined as date of diagnosis (i.e. first HCV positive test or case report) for HCV positive individuals, and date of last negative test result for HCV negative individuals

^d^Mental health, drug misuse, alcohol misuse data was available up to 2012

### Seroconversion and characteristics of new and prevalent HCV cases

Of 370,741 individuals who tested for anti-HCV multiple times since 1992, 7726 (2.1 %) seroconverted. Among the 346,428 currently alive repeat testers, 6922 (2.0 %) seroconverted. Among those testing only once (*n* = 762,114), 56,908 (7.5 %) tested positive (prevalent HCV infections). Of these, 695,137 were alive at the end of 2013 and 46,262 (6.7 %) were positive.

Of the 6922 currently alive seroconverters, 1989 (28.7 %) seroconverted within 12 months, 1497 (21.6 %) in 12-24 months and 3436 (49.6 %) >24-months after their last negative test (Additional file 1: Table S2).

24-month seroconverters and reported acute cases were younger than both >24-month seroconverters and individuals with prevalent infection (median ages: 30, 35, 42 years; *p* < 0.001). Most of the individuals with prevalent infection (66.0 %) were born between 1945-64 with another 19.5 % between 1965-74. Of 24-month seroconverters, 32.4 % were born in 1965-74, and 45.3 % after 1975. A higher percentage of prevalent cases and >24-month seroconverters were male compared to 24-month seroconverters (65.6 %, 59.8 % vs 52.4 %) (Table 2).

**Fig. 1:**
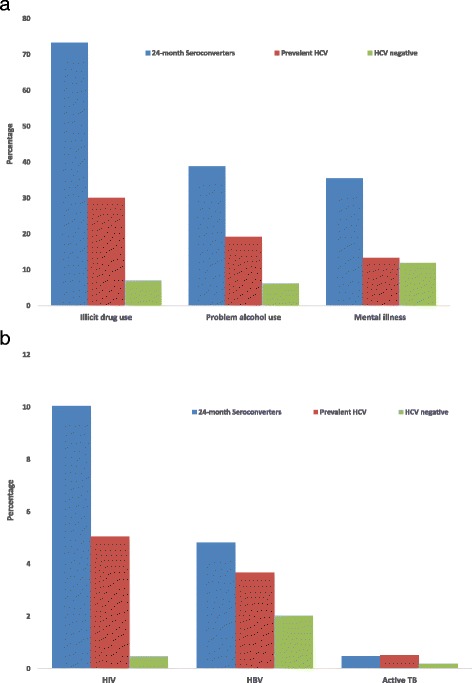
**a** Distribution of illicit drug use, alcohol use, and mental illness by HCV infection status in BC-HTC, Canada 1990-2013. **b** Distribution of co-infections by HCV infection status in BC-HTC, Canada 1990-2013

HIV and HBV co-infections were higher among 24-month seroconverters compared to prevalent HCV cases (HIV: 10.0 % vs. 5.1 %; HBV: 4.8 % vs. 3.7 %) while the proportion with active TB was the same (0.5 % vs. 0.5 %) (Fig. 1a). The proportions with illicit drug use (73.3 % vs. 30.1 %), problem alcohol use (38.9 % vs. 19.2 %) and mental health conditions (35.5 % vs. 13.3 %) were also higher among 24-month seroconverters than individuals with prevalent HCV infection (Table 2, Fig. 1b).

Characteristics of participants diagnosed in recent years (2010-2013) are presented in Additional file 1: Table S3. Overall characteristics are similar with differences in age at diagnosis, birth cohort, HIV, HBV infection, and mental illness mainly in 24 month seroconverters.

### HCV infection by birth cohort

**Fig. 2:**
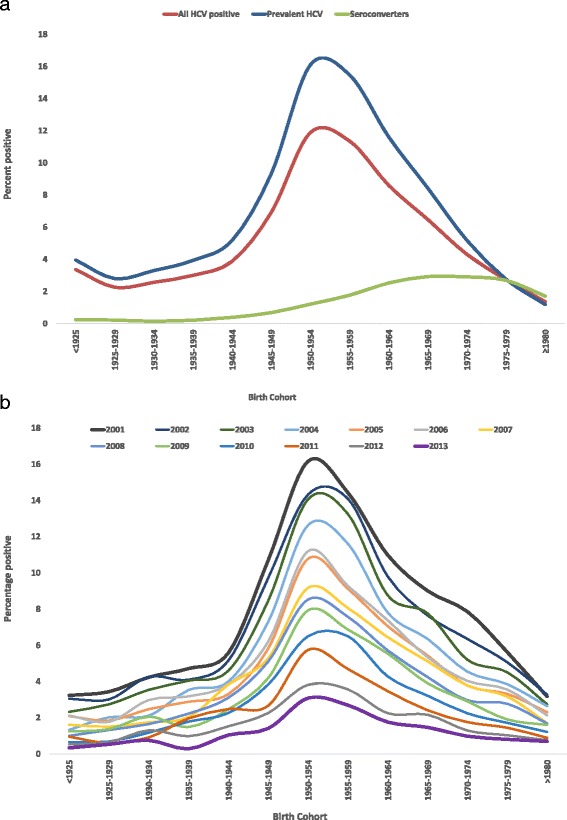
**a** Hepatitis C positivity by birth cohort in BC-HTC, British Columbia, Canada 1990-2013. HCV positives include both seroconverters and chronic HCV. **b** Hepatitis C positivity by birth cohort and year in BC-HTC, British Columbia, Canada, 2001-2013

HCV positivity rates showed a strong birth cohort effect (Fig. 2a, b Additional file 1: Figure S1). Birth cohorts 1950-1954 and 1955-59 had the highest percentage positive overall followed by 1960-64 though positivity declined over time and was lowest in 2013. Birth cohorts 1945-49 and 1965-69 had similar percentage positive though it was slightly higher for 1945-49. In 2013, there were 1,262,121 people born between 1945 and 1964 living in BC; 344,791 (27.3 %) have been tested and 33,946 (9.8 %) were HCV positive, of whom 7352 (17.8 %) have already died. Assuming constant in and outflow, about 70 % of 1945-64 and 1950-70 birth cohorts have not been tested for HCV.

In birth cohort 1945-64, 67.8 % were male compared to 63.9 % and 51.9 % of those born 1965-74 and after 1975, respectively. A small proportion (2.3 %) of 24-month seroconverters was identified among HCV positives born 1945-64 compared to 9.9 % and 24.3 % among 1965-1974 and ≥1975 birth cohorts, respectively. In contrast, 97.9 %, 94.7 %, 79.9 % and 58.8 % HCV positive tests were detected on the first test (prevalent infections) among those born <1945, 1945-64, 1965-1974, and ≥1975, respectively (Table 3).

**Table 3:** Characteristics of currently alive HCV testers by HCV status and birth cohort, Canada, BC-HTC, 1990-2013

	HCV Positive				HCV Negative			
	<1945	1945-1964	1965-1974	≥1975	<1945	1945-1964	1965-1974	≥1975
	*N* = 3168	*N* = 33946	*N* = 11883	*N* = 6784	*N* = 95848	*N* = 310845	*N* = 209956	*N* = 371732
	N (%)	N (%)	N(%)	N (%)	N (%)	N(%)	N (%)	N (%)
Age at baseline^a^								
< 15	0	0	0	438 (6.5)	0	0	0	20289 (5.5)
15-24	0	0	857 (7.2)	2925 (43.1)	0	0	3591 (1.7)	141735 (38.1)
25-34	0	2764 (8.1)	6688 (56.3)	3166 (46.7)	0	6369 (2.1)	66420 (31.6)	186558 (50.2)
35-44	0	14198 (41.8)	4014 (33.8)	255 (3.8)	0	73286 (23.6)	124190 (59.2)	23150 (6.2)
45-54	425 (13.4)	13116 (38.6)	324 (2.7)	0	3166 (3.3)	144213 (46.4)	15755 (7.5)	0
≥ 55	2743 (86.6)	3868 (11.4)	0	0	92682 (96.7)	86977 (28.0)	0	0
Median [IQR]	63 [58-70]	45 [39-50]	32 [28-37]	25 [21–29]	69 [63-75]	50 [44-55]	37 [33-41]	26 [21–30]
Age at 1st test								
< 15	0	0	0	605 (8.9)	0	0	0	25860 (7)
15-24	0	0	1205 (10.1)	3830 (56.5)	0	0	7867 (3.8)	181880 (48.9)
25-34	0	3074 (9.1)	7174 (60.4)	2182 (32.2)	0	12382 (4.0)	96703 (46.1)	151971 (40.9)
35-44	0	14485 (42.7)	3255 (27.4)	167 (2.5)	0	99462 (32.0)	96915 (46.2)	12021 (3.2)
45-54	436 (13.8)	12742 (37.5)	249 (2.1)	0	5217 (5.4)	134000 (43.1)	8471 (4.0)	0
≥ 55	2732 (86.2)	3645 (10.7)	0	0	90631 (94.6)	65001 (20.9)	0	0
Median [IQR]	63 [58-69]	44 [39-50]	31 [27-36]	22 [18–27]	67 [61-73]	48 [42-53]	35 [30-39]	24 [20–28]
Sex								
Female	1354 (42.7)	10946 (32.3)	4288 (36.1)	3261 (48.1)	51063 (53.3)	164778 (53.0)	116339 (55.4)	219772 (59.1)
Male	1809 (57.1)	23000 (67.8)	7594 (63.9)	3523 (51.9)	44657 (46.6)	146061 (47.0)	93615 (44.6)	151954 (40.9)
Unknown	5 (0.2)	0	0	0	128 (0.1)	6 (0.0)	2 (0.0)	6 (0.0)
Year of diagnosis								
1990-1994	340 (10.7)	2938 (8.7)	690 (5.8)	99 (1.5)	822 (0.9)	1858 (0.6)	889 (0.4)	344 (0.1)
1995-1999	1186 (37.4)	12980 (38.2)	3992 (33.6)	857 (12.6)	21815 (22.8)	48746 (15.7)	21003 (10.0)	13299 (3.6)
2000-2004	703 (22.2)	8333 (24.6)	3163 (26.6)	1650 (24.3)	19159 (20.0)	68035 (21.9)	43673 (20.8)	42021 (11.3)
2005-2009	555 (17.5)	6004 (17.7)	2588 (21.8)	2257 (33.3)	22709 (23.7)	81513 (26.2)	63756 (30.4)	104908 (28.2)
2010-2013	384 (12.1)	3691 (10.9)	1450 (12.2)	1921 (28.3)	31343 (32.7)	110693 (35.6)	80635 (38.4)	211160 (56.8)
HIV/AIDS at baseline								
No/Unknown	3149 (99.4)	33150 (97.7)	11364 (95.6)	6562 (96.7)	95664 (99.8)	308950 (99.4)	208736 (99.4)	370832 (99.8)
Yes	19 (0.6)	796 (2.3)	519 (4.4)	222 (3.3)	184 (0.2)	1895 (0.6)	1220 (0.6)	900 (0.2)
HIV/AIDS co-infection (ever)								
No/Unknown	3135 (99.0)	32345 (95.3)	10810 (91.0)	6335 (93.4)	95640 (99.8)	308728 (99.3)	208587 (99.4)	370739 (99.7)
Yes	33 (1.0)	1601 (4.7)	1073 (9.0)	449 (6.6)	208 (0.2)	2117 (0.7)	1369 (0.7)	993 (0.3)
HBV at baseline								
No/Unknown	3130 (98.8)	33529 (98.8)	11686 (98.3)	6729 (99.2)	94660 (98.8)	304567 (98.0)	206304 (98.3)	368692 (99.2)
Yes	38 (1.2)	417 (1.2)	197 (1.7)	55 (0.8)	1188 (1.2)	6278 (2.0)	3652 (1.7)	3040 (0.8)
HBV co-infection (ever)								
No/Unknown	3075 (97.1)	32643 (96.2)	11330 (95.4)	6658 (98.1)	94059 (98.1)	302130 (97.2)	204856 (97.6)	367414 (98.8)
Yes	93 (2.9)	1303 (3.8)	553 (4.7)	126 (1.9)	1789 (1.9)	8715 (2.8)	5100 (2.4)	4318 (1.2)
Active TB at baseline								
No/Unknown	3157 (99.7)	33890 (99.8)	11863 (99.8)	6773 (99.8)	95584 (99.7)	310307 (99.8)	209650 (99.9)	371368 (99.9)
Yes	11 (0.4)	56 (0.2)	20 (0.2)	11 (0.2)	264 (0.3)	538 (0.2)	306 (0.2)	364 (0.1)
Active TB co-infection (ever)								
No/Unknown	3149 (99.4)	33783 (99.5)	11808 (99.4)	6752 (99.5)	95494 (99.6)	310145 (99.8)	209558 (99.8)	371276 (99.9)
Yes	19 (0.6)	163 (0.5)	75 (0.6)	32 (0.5)	354 (0.4)	700 (0.2)	398 (0.2)	456 (0.1)
Material deprivation quintile at baseline								
Unknown	106 (3.4)	1298 (3.8)	618 (5.2)	469 (6.9)	1199 (1.3)	3720 (1.2)	2671 (1.3)	7183 (1.9)
Q1 (most privileged)	507 (16.0)	4444 (13.1)	1362 (11.5)	830 (12.2)	18845 (19.7)	65327 (21.0)	48322 (23.0)	81513 (21.9)
Q2	513 (16.2)	5404 (15.9)	1636 (13.8)	936 (13.8)	18165 (19.0)	60803 (19.6)	39355 (18.7)	69264 (18.6)
Q3	533 (16.8)	6114 (18)	1926 (16.2)	1065 (15.7)	19060 (19.9)	61814 (19.9)	40022 (19.1)	71369 (19.2)
Q4	660 (20.8)	7365 (21.7)	2691 (22.7)	1514 (22.3)	20032 (20.9)	61824 (19.9)	40792 (19.4)	74468 (20.0)
Q5 (most deprived)	849 (26.8)	9321 (27.5)	3650 (30.7)	1970 (29.0)	18547 (19.4)	57357 (18.5)	38794 (18.5)	67935 (18.3)
Social deprivation quintile at baseline								
Unknown	106 (3.4)	1298 (3.8)	618 (5.2)	469 (6.9)	1199 (1.3)	3720 (1.2)	2671 (1.3)	7183 (1.9)
Q1 (most privileged)	557 (17.6)	3477 (10.2)	1081 (9.1)	741 (10.9)	17348 (18.1)	57695 (18.6)	36550 (17.4)	68621 (18.5)
Q2	513 (16.2)	4335 (12.8)	1238 (10.4)	794 (11.7)	17802 (18.6)	56839 (18.3)	36109 (17.2)	64348 (17.3)
Q3	516 (16.3)	5802 (17.1)	1790 (15.1)	989 (14.6)	17342 (18.1)	56864 (18.3)	36204 (17.2)	65305 (17.6)
Q4	606 (19.1)	7152 (21.1)	2380 (20.0)	1257 (18.5)	19201 (20.0)	62437 (20.1)	42960 (20.5)	76953 (20.7)
Q5 (most deprived)	870 (27.5)	11882 (35.0)	4776 (40.2)	2534 (37.4)	22956 (24.0)	73290 (23.6)	55462 (26.4)	89322 (24.0)
Mental illness at baseline^b^								
No/Unknown	2768 (89.8)	28051 (85.0)	9468 (81.9)	4942 (79.5)	75892 (90.3)	230429 (85.6)	160387 (87.7)	262690 (89.8)
Yes	316 (10.2)	4956 (15.0)	2087 (18.1)	1277 (20.5)	8130 (9.7)	38636 (14.4)	22397 (12.3)	29891 (10.2)
Mental illness 3yr pre- baseline^b^								
No/Unknown	2908 (94.3)	30114 (91.2)	10261 (88.8)	5478 (88.1)	80387 (95.7)	251001 (93.3)	172141 (94.2)	277513 (94.8)
Yes	176 (5.7)	2893 (8.8)	1294 (11.2)	741 (11.9)	3635 (4.3)	18064 (6.7)	10643 (5.8)	15068 (5.2)
Illicit drug use at baseline^b^								
No/Unknown	2763 (89.6)	22649 (68.6)	6220 (53.8)	3256 (52.4)	81535 (97.0)	249677 (92.8)	167729 (91.8)	271558 (92.8)
Yes	321 (10.4)	10358 (31.4)	5335 (46.2)	2963 (47.6)	2487 (3.0)	19388 (7.2)	15055 (8.2)	21023 (7.2)
Illicit drug use 3yr pre- baseline^b^								
No/Unknown	2870 (93.1)	25193 (76.3)	7090 (61.4)	3557 (57.2)	83133 (98.9)	259681 (96.5)	174045 (95.2)	278466 (95.2)
Yes	214 (6.9)	7814 (23.7)	4465 (38.6)	2662 (42.8)	889 (1.1)	9384 (3.5)	8739 (4.8)	14115 (4.8)
Problematic alcohol use at baseline^b^								
No/Unknown	2724 (88.3)	25720 (77.9)	8780 (76.0)	4933 (79.3)	79838 (95.0)	248826 (92.5)	171200 (93.7)	277407 (94.8)
Yes	360 (11.7)	7287 (22.1)	2775 (24.0)	1286 (20.7)	4184 (5.0)	20239 (7.5)	11584 (6.3)	15174 (5.2)
Problematic alcohol use 3yr pre-baseline^b^								
No/Unknown	2870 (93.1)	28620 (86.7)	9874 (85.5)	5421 (87.2)	81897 (97.5)	258911 (96.2)	177020 (96.8)	284664 (97.3)
Yes	214 (6.9)	4387 (13.3)	1681 (14.5)	798 (12.8)	2125 (2.5)	10154 (3.8)	5764 (3.2)	7917 (2.7)

^a^Baseline is defined as date of diagnosis (i.e. first HCV positive test or case report) for HCV positive individuals, and date of last negative test result for HCV negative individuals

^b^Mental health, drug misuse, alcohol misuse data was available up to 2012

The proportion of HCV/HIV co-infected individuals increased with increasing birth year (<1945: 1.0 %, 1945-64: 4.7 %, 1965-1974: 9.0 % and >1975: 6.6 %). In contrast, among HCV negative individuals, the HIV infection rates by birth cohort were higher among those born between 1945-64 and 1965-74. HBV co-infection was 2.9 %, 3.8 %, 4.7 % and 1.9 % among those born <1945, 1945-64, 1965-1974, and ≥1975, respectively. Illicit drug use (36.7 % vs 10.4 %), problematic alcohol use (22.3 % vs 11.7 %) and mental health conditions (16.4 % vs 10.2 %) were higher among those born after 1945 than those born before 1945 (Table 3).

### Factors associated with new and prevalent HCV infection

In the multivariable multinomial logistic regression model (Table 4), new HCV infections were associated with being male, born after 1945, HIV co-infection, HBV co-infection, material deprivation, illicit drug use, problematic alcohol use and mental health diagnoses. The same factors were associated with prevalent HCV infections except for lower odds for HBV co-infection, mental health conditions and birth cohort ≥ 1975. The odds of prevalent infection were highest in the 1945-54 birth cohort (OR: 3.8, 95 % CI: 3.7-3.9) followed by 1955-64 (OR: 3.2, 95 % CI: 3.1-3.3), while the odds of being a new infection were highest for the 1975-84 (OR: 11.8, 95 % CI: 9.9-14.1) birth cohort followed by those born 1965-74 (OR: 9.9, 95 % CI: 8.3-11.8). Furthermore, odds ratios for illicit drug use (ORs: 21.0, 95 % CI: 19.8-22.4 vs. 5.0, 95 % CI: 4.9-5.2) and HIV co-infection (ORs: 7.2, 95 % CI: 7.1-8.9 vs. 4.8, 95 % CI: 4.5-5.2) were much higher for new infections than for prevalent HCV infections. In a model with age at diagnosis instead of birth cohort (Additional file 1: Table S5), odds of prevalent infection were highest among ages 45-54 years (OR: 4.0; 95 % CI: 3.9-4.2) followed by 35-44 years (OR: 3.2; 95 % CI: 3.0-3.3) while odds for new infection were highest among ages 15-24 years (OR: 11.4, 95 % CI: 9.0-14.4) and 25-34 years (OR: 11.1, 95 % CI: 8.8-14.0) and declined thereafter. Similar results were observed using indicators for recent risk activities (Additional file 1: Table S6).

**Table 4:** Multivariable multinomial logistic regression models for factors associated with seroconversion and prevalent HCV infection in BC-HTC, Canada 1990-2012^a, b^

Variables	Seroconverters	Prevalent HCV
	ORs (95 % CI)	ORs (95 % CI)
Sex		
Female	1.00	1.00
Male	1.25 (1.19-1.31)	2.06 (2.02-2.10)
Birth year		
< 1945	1.00	1.00
1945-1954	3.38 (2.78-4.11)	3.79 (3.67-3.92)
1955-1964	6.22 (5.19-7.44)	3.23 (3.13-3.34)
1965-1974	9.86 (8.26-11.78)	1.64 (1.58-1.70)
1975-1984	11.76 (9.83-14.07)	0.70 (0.67-0.73)
≥ 1985	6.53 (5.34-7.99)	0.48 (0.44-0.51)
HIV infection at baseline^c^		
No/Unknown	1.00	1.00
Yes	7.92 (7.05-8.89)	4.80 (4.47-5.15)
HBV at baseline^c^		
No/Unknown	1.00	1.00
Yes	2.33 (2.00-2.71)	0.69 (0.63-0.75)
Active TB at baseline^c^		
No/Unknown	1.00	1.00
Yes	1.00 (0.62-1.62)	1.01 (0.81-1.25)
Material deprivation quintile at baseline^c^		
Unknown	3.51 (2.96-4.17)	5.38 (5.07-5.70)
Q1 (most privileged)	1.00	1.00
Q2	1.20 (1.06-1.31)	1.22 (1.18-1.27)
Q3	1.24 (1.13-1.36)	1.36 (1.32-1.41)
Q4	1.62 (1.48-1.76)	1.60 (1.55-1.65)
Q5 (most deprived)	1.99 (1.83-2.15)	1.98 (1.92-2.04)
Mental illness at baseline^c^		
No/Unknown	1.00	1.00
Yes	1.18 (1.12-1.25)	0.69 (0.67-0.72)
Illicit drugs use at baseline		
No/Unknown	1.00	1.00
Yes	21.00 (19.80-22.38)	5.03 (4.91-5.16)
Problematic alcohol use at baseline		
No/Unknown	1.00	1.00
Yes	2.11 (2.00-2.23)	1.67 (1.63-1.72)

^a^Adjusted for health authority and year of diagnosis; ^b^Excluding unknown gender and health authority. ^c^Baseline is defined as time of diagnosis for HCV positive, and date of last negative for HCV negative

## Discussion

We used a large cohort of more than one million people tested for HCV in BC, and while accounting for mortality, we found that a large number of people are currently living with HCV. The highest positivity rate was among people born in 1945-64, which declined over time and was lowest in 2013. New infections were detected mainly among younger age groups. The new HCV infection (seroconversion) rate was highest among males, those with HIV or HBV co-infection, mental health conditions, problematic alcohol or illicit drug use, and socioeconomically disadvantaged persons. Prevalent HCV infection was associated with being male, born 1945-64, HIV co-infected, problem alcohol and illicit drug use, and socioeconomic deprivation. These findings of twin epidemics highlight important opportunities for prevention, testing and treatment of HCV.

In this analysis, we identified two groups of HCV infected individuals: new infections (seroconverters) and those with prevalent HCV infection. Most of those with prevalent HCV were born 1945-1964, while most newly infected individuals were younger. However, the seroconverters are not a homogeneous group: 24-month seroconverters differed from >24 months seroconverters in terms of age, sex, testing patterns, and HBV co-infection rate. Surveillance data on acute HCV infections indicate that 70 % of seroconverters reported injection drug use in the past 12 months [18]. In a BC-HTC subset for whom self-reported risk factor data was available, 85 % of those who self-reported injection drug use had a medical visit for illicit drug use. Thus, 24-month seroconverters most likely acquired HCV through their injection drug use networks.

Most of the HCV cases, especially prevalent infections, were identified among those born 1945-64 and the positivity rate was much higher in this group, especially for those born 1950-1960. Similarly high 1945-64 birth cohort positivity rates have been reported in various studies from the United States [19–22]. Using reported HCV cases in Canada, Trubnikov et al. found the highest HCV prevalence among the 1950-54 and 1955-59 birth cohorts, followed by 1960-64 and 1966-69, while prevalence among those born 1945-49 was lower than for those born 1965-69 [2]. In contrast, in our study, the positivity rate for birth cohort 1945-49 was higher than for birth cohort 1965-69 (Fig. 2). Our findings are also consistent with a study which assessed HCV related hospital admissions where the 1950-54 and 1955-59 birth cohorts had the highest rates [23]. Although the positivity rate is still highest among the 1945-64 birth cohort, the rate in this cohort has been declining over successive years suggesting a decreasing pool of undiagnosed prevalent HCV infections. Canada has had risk based testing guidelines since 1994 [24]. The declining positivity rate in this birth cohort suggests that testing driven by risk based guidelines have been able to identify most people with current or past risk activities and diagnose most of the HCV infections in this cohort. However, risk based testing may not identify individuals who are unable to recall or are unwilling to disclose remote risk behaviors. The US Preventive Services Task Force has recommended one time testing of individuals born 1945-65 [22]. Further studies on feasibility and cost effectiveness of various strategies are needed to identify undiagnosed infections in BC.

Risk factors for both new and prevalent HCV infections were similar with some notable exceptions, including age, birth cohort, drug use, HBV co-infection and mental health diagnoses. HBV co-infection and mental health problems were significantly associated with increased odds of new infection but decreased odds of prevalent HCV infection, while ORs for illicit drug use compared to no drug use among new infections (AOR = 21) were four times than those for prevalent HCV infection (AOR = 5.0). ORs for prevalent HCV infection were highest for birth cohort 1945-64 and declined thereafter, while among new infections, ORs were highest for those born later, consistent with an earlier analysis demonstrating a higher HCV incidence rate in younger birth cohorts [25]. Demographic characteristics and risk factors for prevalent HCV were also consistent with recent data from the United States and Canada [8, 20]. Likewise, in a recent electronic medical record based study from the US, being a baby boomer, male, people who injects drugs (PWID), HIV co-infected and having low income were associated with HCV positivity [26]. Risk factors among new infections were also similar to those identified with HCV infection in high risk populations, mainly PWID. Among seroconverters especially 24-months seroconverters, those born 1965-84, HIV/HBV co-infection, socioeconomic deprivation, mental illness, illicit drug use and problematic alcohol use were more common than among prevalent HCV infections (Table 1/Fig. 1). These findings highlight the presence of syndemics of blood-borne infections (BBI), mental illness, addiction, and socioeconomic marginalization in this population as has been reported by others [27–29]. Addressing these syndemics requires comprehensive services including integrated testing, prevention and treatment for STI/BBIs, as well as mental health and addiction services to address the needs of this population group.

Findings from this paper should be interpreted in the context of some methodological issues. The validity of our estimates depends on the successful linkage rate. Linkage rates were very high for HCV (>85 %), especially in recent years [14]. Linkage rates for those HIV co-infected were much higher than the overall HIV linkage rate, especially before 2005. Thus, the HIV co-infection rate may have been underestimated. However, as reported earlier, we used multiple sources of HIV status identification to reduce underestimation. In this study, we did not have access to immigration and Aboriginal status data and hence we were not able to characterize the disease burden among immigrants and Aboriginal populations. Other data suggest that Aboriginal people are five times more likely to be infected with HCV [30]. Immigrants from endemic countries are also more likely to have higher HCV infection rates.

To date, laboratory testing for HCV has followed risk based guidelines and hence a higher positivity rate among HCV testers in this cohort is expected compared to the general population. However, general population surveys such as the Canadian Health Measures Survey may not capture high risk populations with higher HCV prevalence thus and may underestimate total population of infected individuals [8, 20]. Categorizing prevalent infections based on being positive at the first test may be an over-estimate as some of these individuals are likely to be recent seroconverters as shown in our recent molecular analysis [31]. The prevalent HCV case detection could be affected by presence of late stage symptomatic disease or survival. Survival may also affect the difference in risk factor profile between prevalent and new cases. In the BC-HTC, data is available on all cohort members many years before cohort initiation to assess risk factors as presented in Table 1. Thus, for all cohort members regardless of first test or diagnosis date, data on risk factors started at the same time, providing ample time to assess risk factors from the available data. Furthermore, after accounting for mortality in the cohort, there was no difference in identified risk factor patterns in models including both currently alive and dead and only those currently who are currently alive. Thus, survival bias is unlikely to explain difference in risk factor profile between prevalent and new infections. However, survival bias is not expected to be completely eliminated especially in early nineties, when people could have died before HCV diagnosis or getting diagnosed because of symptoms related to late stage disease. In another analysis, we found that late HCV diagnosis in relation to advance stage liver disease (hepatocellular carcinoma and decompensated cirrhosis) was common in early nineties and have declined substantially over time [32].

In the current paper, data on RNA testing and active infection was not presented, which is important to assess people living with active infection and need treatment to prevent progressive liver disease. The BC-HTC provides a platform to assess program progress through cascade of care monitoring, long term outcomes related to HCV and impact of treatment on long term outcomes. These data are in the process of being analyzed and will be presented in future reports.

## Conclusions

The HCV positivity rate was highest in the 1950-54 and 1955-59 birth cohorts and overall among those born between 1945- 1964, which declined over time. Furthermore, the year over year decline in the positivity rate suggests that most of the HCV infections in these cohorts have already been identified. However, current risk based testing may not identify individuals who are unable to recall or are unwilling to disclose remote risk behaviors. Further studies are needed to estimate the number of undiagnosed HCV infection and assess optimal strategies to identify the remaining undiagnosed infections. Newly acquired infections are occurring mainly in younger birth cohorts and these groups are more likely to be co-infected with HIV and/or HBV, socioeconomically marginalized, and living with serious mental illnesses and addictions. Comprehensive syndemic approaches that take into account co-infections, mental health, additions and socioeconomic vulnerabilities are urgently required to identify, treat, and support people with HCV infection.

## Abbreviations

BC-HTC, The BC Hepatitis Testers Cohort; HCV, hepatitis C virus; BC, British Columbia; HIV, Human immunodeficiency virus; HBV, hepatitis B virus; TB, Tuberculosis; STIs, Sexually transmitted infections; BCCDC, British Columbia Center for Disease Control; BBI, Bloodborne infection; PWID, People who injection drugs

## Additional file

Additional file 1: Table S1. Definitions for comorbid conditions. **Table S2.** Distribution HCV seroconverters by seroconversion interval in BC HTC, Canada 1990-2013. **Table S3.** Characteristics of currently alive recently diagnosed (2010-2013) HCV positive and negative individuals, BC-HTC, 2010 - 2013. **Table S4.** Percentage positive for hepatitis C by diagnosis year and birth cohort in British Columbia, 1992-2013. **Figure S1.** Hepatitis C percentage positive by year of diagnosis and birth cohort, BC-HTC, British Columbia, Canada, 2000-2013. **Table S5.** Multivariable multinomial logistic regression model for factors associated with seroconversion and chronic HCV infection including age as covariate in BC HTC, Canada 1990-2012 a,b. **Table S6.** Multivariable multinomial logistic regression model for factors associated with seroconversion and chronic HCV infection including indicators for recent risk activities in BC HTC, Canada 1990-2012 a,b. (DOCX 68 kb)
