# Supplementary material for: Twin epidemics of new and prevalent hepatitis C infections in Canada: BC Hepatitis Testers Cohort
Source: BMC Infect Dis. 2016 Jul 19;16:334. doi: 10.1186/s12879-016-1683-z (PMC4952323; doi:10.1186/s12879-016-1683-z)
Supplement: Additional file 1: Table S1. — Definitions for comorbid conditions. Table S2. Distribution HCV seroconverters by seroconversion interval in BC HTC, Canada 1990-2013. Table S3. Characteristics of currently alive recently diagnosed (2010-2013) HCV positive and negative individuals, BC-HTC, 2010 - 2013. Table S4. Percentage positive for hepatitis C by diagnosis year and birth cohort in British Columbia, 1992-2013. Figure S1. Hepatitis C percentage positive by year of diagnosis and birth cohort, BC-HTC, British Columbia, Canada, 2000-2013. Table S5. Multivariable multinomial logistic regression model for factors associated with seroconversion and chronic HCV infection including age as covariate in BC HTC, Canada 1990-2012 a,b. Table S6. Multivariable multinomial logistic regression model for factors associated with seroconversion and chronic HCV infection including indicators for recent risk activities in BC HTC, Canada 1990-2012 a,b. (DOCX 68 kb) [file 12879_2016_1683_MOESM1_ESM.docx]

**Online Supplement**

**Supplement Table 1. Definitions for comorbid conditions**

| **1. Mental Illness**  Major mental illness was flagged at the first occurrence of either 1 hospitalization diagnostic code OR 2 MSP diagnostic codes from a psychiatrist visit for schizophrenic, bipolar, delusional, nonorganic psychotic, adjustment, anxiety, dissociative, personality and major depressive disorders  Physician Billing Data: MSP ICD-9 diagnostic codes: starting with 295-298, 300-301, 308-309, 311 AND claim specialty = 3  Hospitalization Data: DAD1/ICD-9-CM: starting with 295-298, 300-301, 308-309, 311; DAD2/ICD-10-CA: starting with F20-F25, F28-F34, F38-F45, F48, F60-F61 |
| --- |
| **2. Problematic Alcohol Use**  Problematic alcohol use was defined at the first occurrence of 1 MSP or 1 hospitalization codes for major alcohol-related diagnoses including alcoholic mental disorders and dependence/abuse syndromes; alcoholic polyneuropathy, myopathy, cardiomyopathy; pseudo Cushing’s syndrome; or discharge to alcohol rehabilitation, counselling, or surveillance  Physician Billing Data: MSP ICD-9 diagnostic codes: starting with 291, 303, 3050, 3575, 4255  Hospitalization Data: DAD1/ICD-9-CM: starting with 291, 303, 3050,3575, 4255; DAD2/ICD-10-CA: starting with F10, E244, G312, G621, G721, I426, Z502, Z714 |
| **3. Illicit Drug Use**  Illicit Drug Use was defined at the first occurrence of 1 MSP or 1 hospitalization diagnostic codes for major drug-related diagnoses involving addiction, dependence, and drug-induced mental disorders; illicit drug use, or illicit use of prescribed drugs including: hallucinogens, barbituates/tranquillizers, sedatives, hypnotics, anxiolytics, opioids, cocaine, amphetamine, volatile solvents; or discharge to drug rehabilitation, counselling, surveillance or methadone/buprenorphine substitution treatment  Physician Billing Data: MSP ICD-9 diagnostic codes: starting with 292, 304, 3053-3057 or exact codes V6542 or 39  Hospitalization Data: DAD1/ICD-9-CM: starting with 292, 304, 3053-3057 or exact code V6542; DAD2/ICD-10-CA: starting with F11-F16, F18-F19, Z715, Z503 |

**Supplement Table 2. Distribution HCV seroconverters by seroconversion interval in BC HTC, Canada 1990-2013**

|  | **Seroconverters** | | | **Prevalent HCV** | **All Positives** |
| --- | --- | --- | --- | --- | --- |
|  | **<12 mon ^a^** | **12- 24 mon ^b^** | **>24 mon ^c^** |  |  |
|  | N(%) | N(%) | N(%) | N(%) | N(%) |
|  | N=2131 | N=1497 | N=3436 | N=48717 | N=55781 |
| **Age at diagnosis (years)** |  |  |  |  |  |
| <15 | 16(0.8) | 1(0.1) | 1(0) | 420(0.9) | 438(0.8) |
| 15-24 | 596(28) | 366(24.5) | 410(11.9) | 2410(5) | 3782(6.8) |
| 25-34 | 845(39.7) | 632(42.2) | 1234(35.9) | 9907(20.3) | 12618(22.6) |
| 35-44 | 462(21.7) | 365(24.4) | 1106(32.2) | 16534(33.9) | 18467(33.1) |
| 45-54 | 171(8) | 104(7) | 522(15.2) | 13068(26.8) | 13865(24.9) |
| ≥55 | 41(1.9) | 29(1.9) | 163(4.7) | 6378(13.1) | 6611(11.9) |
| Median[IQR] | 29[24 - 37] | 30[25 - 37] | 35[28 - 43] | 42[34 - 49] | 41[33 - 48] |
| **Age at 1st test** |  |  |  |  |  |
| <15 | 68(3.2) | 32(2.1) | 84(2.4) | 421(0.9) | 605(1.1) |
| 15-24 | 865(40.6) | 620(41.4) | 1128(32.8) | 2422(5) | 5035(9) |
| 25-34 | 692(32.5) | 533(35.6) | 1278(37.2) | 9927(20.4) | 12430(22.3) |
| 35-44 | 369(17.3) | 236(15.8) | 712(20.7) | 16590(34.1) | 17907(32.1) |
| 45-54 | 106(5) | 63(4.2) | 195(5.7) | 13063(26.8) | 13427(24.1) |
| ≥55 | 31(1.5) | 13(0.9) | 39(1.1) | 6294(12.9) | 6377(11.4) |
| Median[IQR] | 26[20 - 34] | 26[20 - 33] | 28[22 - 35] | 42[34 - 49] | 40[32 - 48] |
| **Birth year** |  |  |  |  |  |
| <1945 | 19(0.9) | 12(0.8) | 35(1) | 3102(6.4) | 3168(5.7) |
| 1945-1964 | 466(21.9) | 310(20.7) | 1037(30.2) | 32133(66) | 33946(60.9) |
| 1965-1974 | 669(31.4) | 507(33.9) | 1214(35.3) | 9493(19.5) | 11883(21.3) |
| ≥1975 | 977(45.9) | 668(44.6) | 1150(33.5) | 3989(8.2) | 6784(12.2) |
| **Sex** |  |  |  |  |  |
| Female | 1052(49.4) | 676(45.2) | 1380(40.2) | 16741(34.4) | 19849(35.6) |
| Male | 1079(50.6) | 821(54.8) | 2056(59.8) | 31970(65.6) | 35926(64.4) |
| Unknown | 0(0) | 0(0) | 0(0) | 6(0) | 6(0) |
| **Year of diagnosis** |  |  |  |  |  |
| 1990-1994 | 67(3.1) | 18(1.2) | 4(0.1) | 3978(8.2) | 4067(7.3) |
| 1995-1999 | 442(20.7) | 284(19) | 353(10.3) | 17936(36.8) | 19015(34.1) |
| 2000-2004 | 691(32.4) | 486(32.5) | 939(27.3) | 11733(24.1) | 13849(24.8) |
| 2005-2009 | 553(26) | 441(29.5) | 1280(37.3) | 9130(18.7) | 11404(20.4) |
| 2010-2013 | 378(17.7) | 268(17.9) | 860(25) | 5940(12.2) | 7446(13.4) |
| **HIV at baseline ^d^** |  |  |  |  |  |
| Unknown | 2014(94.5) | 1436(95.9) | 3207(93.3) | 47568(97.6) | 54225(97.2) |
| Yes | 117(5.5) | 61(4.1) | 229(6.7) | 1149(2.4) | 1556(2.8) |
| **HIV co-infection (ever)** | |  |  |  |  |
| Unknown | 1894(88.9) | 1370(91.5) | 3104(90.3) | 46257(95) | 52625(94.3) |
| Yes | 237(11.1) | 127(8.5) | 332(9.7) | 2460(5.1) | 3156(5.7) |
| **HBV at baseline ^d^** |  |  |  |  |  |
| Unknown | 2074(97.3) | 1454(97.1) | 3349(97.5) | 48197(98.9) | 55074(98.7) |
| Yes | 57(2.7) | 43(2.9) | 87(2.5) | 520(1.1) | 707(1.3) |
| **HBV co-infection (ever)** | |  |  |  |  |
| Unknown | 2028(95.2) | 1425(95.2) | 3326(96.8) | 46927(96.3) | 53706(96.3) |
| Yes | 103(4.8) | 72(4.8) | 110(3.2) | 1790(3.7) | 2075(3.7) |
| **Active TB at baseline ^d^** | |  |  |  |  |
| Unknown | 2129(99.9) | 1492(99.7) | 3428(99.8) | 48633(99.8) | 55682(99.8) |
| Yes | 2(0.1) | 5(0.3) | 8(0.2) | 84(0.2) | 99(0.2) |
| **Active TB co-infection (ever)** | |  |  |  |  |
| Unknown | 2122(99.6) | 1489(99.5) | 3415(99.4) | 48458(99.5) | 55484(99.5) |
| Yes | 9(0.4) | 8(0.5) | 21(0.6) | 259(0.5) | 297(0.5) |
| **Material deprivation quintile** |  |  |  |  |  |
| Unknown | 70(3.3) | 26(1.7) | 79(2.3) | 2316(4.8) | 2491(4.5) |
| Q1 (most privileged) | 263(12.3) | 189(12.6) | 444(12.9) | 6247(12.8) | 7143(12.8) |
| Q2 | 324(15.2) | 202(13.5) | 463(13.5) | 7500(15.4) | 8489(15.2) |
| Q3 | 320(15) | 211(14.1) | 585(17) | 8522(17.5) | 9638(17.3) |
| Q4 | 515(24.2) | 361(24.1) | 774(22.5) | 10580(21.7) | 12230(21.9) |
| Q5 (most deprived) | 639(30) | 508(33.9) | 1091(31.8) | 13552(27.8) | 15790(28.3) |
| **Social deprivation quintile** |  |  |  |  |  |
| Unknown | 70(3.3) | 26(1.7) | 79(2.3) | 2316(4.8) | 2491(4.5) |
| Q1 (most privileged) | 160(7.5) | 111(7.4) | 283(8.2) | 5302(10.9) | 5856(10.5) |
| Q2 | 219(10.3) | 130(8.7) | 337(9.8) | 6194(12.7) | 6880(12.3) |
| Q3 | 312(14.6) | 228(15.2) | 463(13.5) | 8094(16.6) | 9097(16.3) |
| Q4 | 426(20) | 277(18.5) | 720(21) | 9972(20.5) | 11395(20.4) |
| Q5 (most deprived) | 944(44.3) | 725(48.4) | 1554(45.2) | 16839(34.6) | 20062(36) |
| **Mental illness at baseline ^d,e^** |  |  |  |  |  |
| No | 1256(62.9) | 943(66.9) | 2096(65) | 40934(86.7) | 45229(84) |
| Yes | 742(37.1) | 467(33.1) | 1130(35) | 6297(13.3) | 8636(16) |
| **Illicit drug use at baseline ^d,e^** |  |  |  |  |  |
| No | 570(28.5) | 340(24.1) | 942(29.2) | 33036(70) | 34888(64.8) |
| Yes | 1428(71.5) | 1070(75.9) | 2284(70.8) | 14195(30.1) | 18977(35.2) |
| **Problem alcohol use at baseline ^d,e^** |  |  |  |  |  |
| No | 1239(62) | 844(59.9) | 1896(58.8) | 38178(80.8) | 42157(78.3) |
| Yes | 759(38) | 566(40.1) | 1330(41.2) | 9053(19.2) | 11708(21.7) |
| **Mental illness 3yr pre baseline ^d,e^** |  |  |  |  |  |
| No | 1493(74.7) | 1097(77.8) | 2653(82.2) | 43518(92.1) | 48761(90.5) |
| Yes | 505(25.3) | 313(22.2) | 573(17.8) | 3713(7.9) | 5104(9.5) |
| **Illicit drug use 3yr pre baseline ^d,e^** |  |  |  |  |  |
| No | 673(33.7) | 411(29.2) | 1433(44.4) | 36193(76.6) | 38710(71.9) |
| Yes | 1325(66.3) | 999(70.9) | 1793(55.6) | 11038(23.4) | 15155(28.1) |
| **Problem alcohol use 3yr pre baseline ^d,e^** |  |  |  |  |  |
| No | 1488(74.5) | 1033(73.3) | 2555(79.2) | 41709(88.3) | 46785(86.9) |
| Yes | 510(25.5) | 377(26.7) | 671(20.8) | 5522(11.7) | 7080(13.1) |

^a^ 12 month sero- Individuals who seroconverted within 12 months of the last negative test;

^b^ 24 month sero: Individuals who seroconverted between 12- 24 months of the last negative test;

^c^ Individuals who seroconverted after 24 months of the last negative test;

^d^ Baseline is defined as date of diagnosis (i.e. first HCV positive test or case report) for HCV positive individuals, and date of last negative test result for HCV negative individuals;

^e^ Mental health, drug misuse, alcohol misuse data was available up to 2012

**Supplement Table 3: Characteristics of currently alive recently diagnosed (2010-2013) HCV positive and negative individuals, BC-HTC, 2010 - 2013**

|  | **HCV +ve group** | | | | **HCV -ve group** |
| --- | --- | --- | --- | --- | --- |
|  | **M24 Sero^a^** | **>M24 Sero ^b^** | **Prevalent HCV** | **All Positives** | **All Negatives** |
|  | N=646 | N=860 | N=5940 | N=7446 | N=433831 |
|  | **N(%)** | **N(%)** | **N(%)** | **N(%)** | **N(%)** |
| **Age at diagnosis (yrs)** |  |  |  |  |  |
| <15 | 3(0.5) | 0(0) | 30(0.5) | 33(0.4) | 5406(1.3) |
| 15-24 | 125(19.4) | 70(8.1) | 215(3.6) | 410(5.5) | 62546(14.4) |
| 25-34 | 265(41) | 267(31.1) | 691(11.6) | 1223(16.4) | 120058(27.7) |
| 35-44 | 149(23.1) | 270(31.4) | 962(16.2) | 1381(18.6) | 88030(20.3) |
| 45-54 | 74(11.5) | 178(20.7) | 1759(29.6) | 2011(27) | 67985(15.7) |
| ≥55 | 30(4.6) | 75(8.7) | 2283(38.4) | 2388(32.1) | 89806(20.7) |
| Median[IQR] | 31[26 - 40] | 38[30 - 46] | 51[41 - 58] | 48[36 - 57] | 37[27 - 53] |
| **Age at 1st test (yrs)** |  |  |  |  |  |
| <15 | 32(5) | 33(3.8) | 30(0.5) | 95(1.3) | 9145(2.1) |
| 15-24 | 290(44.9) | 288(33.5) | 216(3.6) | 794(10.7) | 93376(21.5) |
| 25-34 | 183(28.3) | 283(32.9) | 698(11.8) | 1164(15.6) | 119400(27.5) |
| 35-44 | 90(13.9) | 188(21.9) | 987(16.6) | 1265(17) | 80115(18.5) |
| 45-54 | 32(5) | 54(6.3) | 1766(29.7) | 1852(24.9) | 58527(13.5) |
| ≥55 | 19(2.9) | 14(1.6) | 2243(37.8) | 2276(30.6) | 73268(16.9) |
| Median[IQR] | 25[19 - 33] | 27.5[21 - 36] | 51[40 - 58] | 47[33 - 56] | 37[27 - 53] |
| **Birth year** |  |  |  |  |  |
| <1945 | 6(0.9) | 8(0.9) | 370(6.2) | 384(5.2) | 31343(7.2) |
| 1945-1964 | 75(11.6) | 193(22.4) | 3423(57.6) | 3691(49.6) | 110693(25.5) |
| 1965-1974 | 124(19.2) | 276(32.1) | 1050(17.7) | 1450(19.5) | 80635(18.6) |
| ≥1975 | 441(68.3) | 383(44.5) | 1097(18.5) | 1921(25.8) | 211160(48.7) |
| **Sex** |  |  |  |  |  |
| Female | 321(49.7) | 352(40.9) | 1958(33) | 2631(35.3) | 241552(55.7) |
| Male | 325(50.3) | 508(59.1) | 3981(67) | 4814(64.7) | 192255(44.3) |
| Unknown | 0(0) | 0(0) | 1(0) | 1(0) | 24(0) |
| **HIV at baseline ^c^** |  |  |  |  |  |
| Unknown | 596(92.3) | 787(91.5) | 5821(98) | 7204(96.8) | 431226(99.4) |
| Yes | 50(7.7) | 73(8.5) | 119(2) | 242(3.3) | 2605(0.6) |
| **HIV (ever)** |  |  |  |  |  |
| Unknown | 591(91.5) | 780(90.7) | 5800(97.6) | 7171(96.3) | 431150(99.4) |
| Yes | 55(8.5) | 80(9.3) | 140(2.4) | 275(3.7) | 2681(0.6) |
| **HBV at baseline ^c^** |  |  |  |  |  |
| Unknown | 640(99.1) | 838(97.4) | 5902(99.4) | 7380(99.1) | 427508(98.5) |
| Yes | 6(0.9) | 22(2.6) | 38(0.6) | 66(0.9) | 6323(1.5) |
| **HBV (ever)** |  |  |  |  |  |
| Unknown | 640(99.1) | 838(97.4) | 5858(98.6) | 7336(98.5) | 426003(98.2) |
| Yes | 6(0.9) | 22(2.6) | 82(1.4) | 110(1.5) | 7828(1.8) |
| **Active TB at baseline ^c^** |  |  |  |  |  |
| Unknown | 643(99.5) | 856(99.5) | 5929(99.8) | 7428(99.8) | 432984(99.8) |
| Yes | 3(0.5) | 4(0.5) | 11(0.2) | 18(0.2) | 847(0.2) |
| **Active TB (ever)** |  |  |  |  |  |
| Unknown | 643(99.5) | 856(99.5) | 5923(99.7) | 7422(99.7) | 432895(99.8) |
| Yes | 3(0.5) | 4(0.5) | 17(0.3) | 24(0.3) | 936(0.2) |
| **Material deprivation quintile** |  |  |  |  |  |
| Unknown | 14(2.2) | 23(2.7) | 540(9.1) | 577(7.8) | 8105(1.9) |
| Q1 (most privileged) | 124(19.2) | 140(16.3) | 808(13.6) | 1072(14.4) | 100533(23.2) |
| Q2 | 92(14.2) | 116(13.5) | 935(15.7) | 1143(15.4) | 84214(19.4) |
| Q3 | 79(12.2) | 126(14.7) | 1020(17.2) | 1225(16.5) | 84376(19.5) |
| Q4 | 117(18.1) | 169(19.7) | 1197(20.2) | 1483(19.9) | 85281(19.7) |
| Q5 (most deprived) | 220(34.1) | 286(33.3) | 1440(24.2) | 1946(26.1) | 71322(16.4) |
| **Social deprivation quintile** |  |  |  |  |  |
| Unknown | 14(2.2) | 23(2.7) | 540(9.1) | 577(7.8) | 8105(1.9) |
| Q1 (most privileged) | 56(8.7) | 84(9.8) | 784(13.2) | 924(12.4) | 81312(18.7) |
| Q2 | 62(9.6) | 80(9.3) | 755(12.7) | 897(12.1) | 76530(17.6) |
| Q3 | 92(14.2) | 112(13) | 895(15.1) | 1099(14.8) | 74989(17.3) |
| Q4 | 123(19) | 181(21.1) | 1171(19.7) | 1475(19.8) | 89537(20.6) |
| Q5 (most deprived) | 299(46.3) | 380(44.2) | 1795(30.2) | 2474(33.2) | 103358(23.8) |
| **Mental illness at baseline ^c,d^** |  |  |  |  |  |
| No | 236(55.4) | 384(59.1) | 3843(86.3) | 4463(80.7) | 236182(86.2) |
| Yes | 190(44.6) | 266(40.9) | 611(13.7) | 1067(19.3) | 37720(13.8) |
| **Mental illness 3yr pre baseline ^d^** |  |  |  |  |  |
| No | 306(71.8) | 520(80) | 4167(93.6) | 4993(90.3) | 257268(93.9) |
| Yes | 120(28.2) | 130(20) | 287(6.4) | 537(9.7) | 16634(6.1) |
| **Illicit drug use at baseline ^c,d^** |  |  |  |  |  |
| No | 112(26.3) | 192(29.5) | 3440(77.2) | 3744(67.7) | 250250(91.4) |
| Yes | 314(73.7) | 458(70.5) | 1014(22.8) | 1786(32.3) | 23652(8.6) |
| **Illicit drug use 3yr pre baseline ^d^** |  |  |  |  |  |
| No | 144(33.8) | 311(47.9) | 3859(86.6) | 4314(78) | 261228(95.4) |
| Yes | 282(66.2) | 339(52.2) | 595(13.4) | 1216(22) | 12674(4.6) |
| **Problem alcohol use at baseline ^c,d^** |  |  |  |  |  |
| No | 251(58.9) | 372(57.2) | 3710(83.3) | 4333(78.4) | 253349(92.5) |
| Yes | 175(41.1) | 278(42.8) | 744(16.7) | 1197(21.7) | 20553(7.5) |
|  |  |  |  |  |  |
| **Problem alcohol use 3yr pre baseline ^d^** |  |  |  |  |  |
| No | 312(73.2) | 524(80.6) | 4096(92) | 4932(89.2) | 264202(96.5) |
| Yes | 114(26.8) | 126(19.4) | 358(8) | 598(10.8) | 9700(3.5) |

^a^ 24-month Sero: Individuals who seroconverted within 24 months of the last negative test;

^b^ Individuals who seroconverted after 24 months of the last negative test;

^c^ Baseline is defined as date of diagnosis (i.e. first HCV positive test or case report) for HCV positive individuals, and date of last negative test result for HCV negative individuals;

^d^ Mental health, drug misuse, alcohol misuse data was available up to 2012

**Supplement Table 4. Percentage positive for hepatitis C by diagnosis year and birth cohort in British Columbia, 1992-2013**

|  | **Birth Cohorts** | | | | | | | | | | | | |
| --- | --- | --- | --- | --- | --- | --- | --- | --- | --- | --- | --- | --- | --- |
| **HCV diagnosis**  **year** | <1925 | 1925-1929 | 1930-1934 | 1935-1939 | 1940-1944 | 1945-1949 | 1950-1954 | 1955-1959 | 1960-1964 | 1965-1969 | 1970-1974 | 1975-1979 | >1980 |
| 1992 | 48.2 | 40.0 | 39.5 | 66.1 | 60.3 | 75.0 | 81.3 | 81.5 | 77.3 | 73.0 | 55.6 | 36.8 | 38.7 |
| 1993 | 29.8 | 24.5 | 32.8 | 35.6 | 38.9 | 58.2 | 70.9 | 69.1 | 60.7 | 48.7 | 41.6 | 20.0 | 13.2 |
| 1994 | 15.7 | 21.7 | 24.4 | 24.5 | 35.8 | 50.7 | 64.8 | 64.2 | 57.8 | 48.6 | 35.2 | 31.3 | 15.8 |
| 1995 | 14.8 | 12.7 | 16.6 | 18.8 | 21.8 | 35.9 | 50.0 | 52.8 | 46.7 | 38.5 | 27.1 | 14.2 | 9.0 |
| 1996 | 7.5 | 8.3 | 8.2 | 9.9 | 13.9 | 26.5 | 37.7 | 35.7 | 30.4 | 25.2 | 19.5 | 9.0 | 5.3 |
| 1997 | 3.6 | 4.3 | 4.3 | 5.5 | 6.6 | 13.6 | 21.7 | 20.1 | 17.7 | 17.3 | 14.3 | 8.3 | 3.1 |
| 1998 | 4.2 | 4.0 | 5.7 | 7.2 | 9.4 | 16.9 | 26.7 | 24.6 | 20.1 | 18.0 | 14.0 | 9.5 | 3.9 |
| 1999 | 3.6 | 4.4 | 5.3 | 6.5 | 8.4 | 15.0 | 23.3 | 20.5 | 15.9 | 11.9 | 11.6 | 7.2 | 3.8 |
| 2000 | 3.1 | 3.6 | 4.4 | 4.7 | 6.8 | 11.4 | 18.6 | 18.2 | 12.7 | 10.3 | 8.5 | 6.9 | 4.0 |
| 2001 | 3.2 | 3.4 | 4.2 | 4.7 | 5.6 | 10.7 | 16.2 | 14.4 | 11.0 | 9.0 | 7.9 | 5.6 | 3.2 |
| 2002 | 3.1 | 3.0 | 4.3 | 4.1 | 5.2 | 9.7 | 14.3 | 14.1 | 9.8 | 7.6 | 6.3 | 5.1 | 3.3 |
| 2003 | 2.3 | 2.7 | 3.5 | 4.0 | 4.6 | 8.5 | 14.1 | 13.3 | 8.8 | 7.8 | 5.2 | 4.5 | 2.7 |
| 2004 | 1.3 | 2.0 | 2.1 | 3.5 | 4.0 | 7.3 | 12.6 | 11.6 | 7.8 | 6.4 | 4.5 | 3.8 | 2.6 |
| 2005 | 2.1 | 1.8 | 2.5 | 2.9 | 3.3 | 5.8 | 10.8 | 9.1 | 7.1 | 5.4 | 3.8 | 3.3 | 2.3 |
| 2006 | 2.1 | 1.8 | 3.0 | 3.2 | 3.8 | 6.2 | 11.2 | 9.3 | 7.4 | 5.3 | 4.0 | 3.6 | 2.1 |
| 2007 | 1.6 | 1.5 | 1.7 | 2.0 | 3.8 | 5.3 | 9.2 | 8.1 | 6.4 | 5.1 | 3.8 | 3.2 | 1.7 |
| 2008 | 1.0 | 1.3 | 1.6 | 2.2 | 3.1 | 5.1 | 8.5 | 7.6 | 5.7 | 4.2 | 3.0 | 2.8 | 1.7 |
| 2009 | 1.2 | 1.4 | 2.1 | 1.5 | 2.5 | 4.2 | 7.9 | 6.9 | 5.5 | 3.9 | 2.9 | 1.9 | 1.6 |
| 2010 | 0.6 | 0.7 | 1.2 | 1.8 | 2.3 | 3.8 | 6.5 | 6.5 | 4.3 | 3.2 | 2.2 | 1.7 | 1.2 |
| 2011 | 1.0 | 0.6 | 0.9 | 2.0 | 2.5 | 2.7 | 5.7 | 4.7 | 3.5 | 2.4 | 1.8 | 1.4 | 0.9 |
| 2012 | 0.5 | 0.6 | 1.3 | 1.0 | 1.5 | 2.3 | 3.8 | 3.6 | 2.2 | 2.2 | 1.3 | 1.0 | 0.8 |
| 2013 | 0.3 | 0.5 | 0.7 | 0.3 | 1.0 | 1.4 | 3.1 | 2.7 | 1.8 | 1.5 | 1.0 | 0.8 | 0.7 |
| Test of trend in HCV positivity |  |  |  |  |  |  |  |  |  |  |  |  |  |
| Linear regression β_Year_ (95%CI) | -0.012  (-0.018,  -0.006) | -0.011  (-0.016,  -0.007) | -0.013  (-0.018,  -0.007) | -0.016  (-0.024,  -0.009) | -0.018  (-0.025,  -0.01) | -0.025  (-0.034,  -0.017) | -0.03  (-0.039,  -0.022) | -0.03  (-0.039,  -0.022) | -0.028  (-0.036,  -0.019) | -0.024  (-0.032,  -0.017) | -0.019  (-0.025,  -0.013) | -0.012  (-0.016,  -0.008) | -0.009  (-0.013,  -0.004) |
| *P- Cochrane trend test* | <0.001 | <0.001 | <0.001 | <0.001 | <0.001 | <0.001 | <0.001 | <0.001 | <0.001 | <0.001 | <0.001 | <0.001 | <0.001 |

**Supplement Figure 1. Hepatitis C percentage positive by year of diagnosis and birth cohort, BC-HTC, British Columbia, Canada, 2000-2013**

**Supplement Table 5.** **Multivariable multinomial logistic regression model for factors associated with seroconversion and chronic HCV infection including age as covariate in BC HTC, Canada 1990-2012 ^a,b^**

| **Variables** | **Seroconverters** | **Chronic HCV** |
| --- | --- | --- |
|  | **ORs(95%CI)** | **ORs(95%CI)** |
| **Sex** |  |  |
| Female | 1.00 | 1.00 |
| Male | 1.27(1.21-1.34) | 2.08(2.04-2.12) |
| **Age (years)** |  |  |
| <15 | 1.90(1.14-3.15) | 0.68(0.61-0.76) |
| 15-24 | 11.36(8.95-14.43) | 0.83(0.79-0.88) |
| 25-34 | 11.10(8.77-14.04) | 1.79(1.71-1.86) |
| 35-44 | 7.68(6.06-9.74) | 3.15(3.02-3.27) |
| 45-54 | 4.68(3.67-5.96) | 4.04(3.88-4.21) |
| 55-64 | 2.58(1.98-3.36) | 2.41(2.31-2.53) |
| ≥65 | 1.00 | 1.00 |
| **HIV infection at baseline ^c^** |  |  |
| No/Unknown | 1.00 | 1.00 |
| Yes | 8.20(7.30-9.20) | 5.00(4.66-5.37) |
| **HBV at baseline ^c^** |  |  |
| No/Unknown | 1.00 | 1.00 |
| Yes | 2.39(2.06-2.78) | 0.72(0.66-0.79) |
| **Active TB at baseline ^c^** |  |  |
| No/Unknown | 1.00 | 1.00 |
| Yes | 0.99(0.61-1.61) | 0.99(0.80-1.24) |
| **Material deprivation quintile at baseline ^c^** |  |  |
| Unknown | 3.58(3.02-4.25) | 5.32(5.02-5.64) |
| Q1 (most privileged) | 1.00 | 1.00 |
| Q2 | 1.20(1.09-1.31) | 1.24(1.19-1.28) |
| Q3 | 1.24(1.13-1.36) | 1.37(1.33-1.42) |
| Q4 | 1.62(1.49-1.76) | 1.60(1.55-1.65) |
| Q5 (most deprived) | 1.99(1.84-2.16) | 2.00(1.93-2.06) |
| **Mental illness at baseline** |  |  |
| No/Unknown | 1.00 | 1.00 |
| Yes | 1.19(1.12-1.25) | 0.70(0.68-0.72) |
| **Illicit drugs use at baseline** |  |  |
| No/Unknown | 1.00 | 1.00 |
| Yes | 20.88(19.69-22.15) | 5.02(4.90-5.15) |
| **Problematic alcohol use at baseline** |  |  |
| No/Unknown | 1.00 | 1.00 |
| Yes | 2.11(2.00-2.23) | 1.69(1.65-1.74) |

a Adjusted for health authority and year of diagnosis; b Excluding unknown gender and health authority. c Baseline is defined as time of diagnosis for HCV positive, and date of last negative for HCV negative

**Supplement Table 6.** **Multivariable multinomial logistic regression model for factors associated with seroconversion and chronic HCV infection including indicators for recent risk activities in BC HTC, Canada 1990-2012 ^a,b^**

| **Variables** | **Seroconverters** | **Chronic HCV** |
| --- | --- | --- |
|  | **ORs(95%CI)** | **ORs(95%CI)** |
| **Sex** |  |  |
| Female | 1.00 | 1.00 |
| Male | 1.23(1.17-1.29) | 2.10(2.06-2.14) |
| **Birth year** |  |  |
| <1945 | 1.00 | 1.00 |
| 1945-1954 | 3.60(2.97-4.38) | 3.86(3.73-3.98) |
| 1955-1964 | 6.45(5.39-7.72) | 3.28(3.18-3.39) |
| 1965-1974 | 9.09(7.61-10.86) | 1.61(1.55-1.67) |
| 1975-1984 | 9.50(7.94-11.37) | 0.66(0.63-0.69) |
| ≥1985 | 4.53(3.70-5.54) | 0.43(0.40-0.46) |
| **HIV infection at baseline** |  |  |
| No/Unknown | 1.00 | 1.00 |
| Yes | 8.99(8.00-10.11) | 5.08(4.73-5.46) |
| **HBV at baseline** |  |  |
| Unknown | 1.00 | 1.00 |
| Yes | 2.21(1.90-2.57) | 0.67(0.61-0.73) |
| **Active TB at baseline** |  |  |
| Unknown | 1.00 | 1.00 |
| Yes | 1.06(0.65-1.72) | 1.07(0.87-1.33) |
| **Material deprivation quintile at baseline** |  |  |
| Unknown | 3.47(2.92-4.12) | 5.37(5.07 -5.70) |
| Q1 (most privileged) | 1.00 | 1.00 |
| Q2 | 1.22(1.11-1.34) | 1.24(1.20-1.28) |
| Q3 | 1.26(1.15-1.39) | 1.38(1.33-1.43) |
| Q4 | 1.66(1.53-1.81) | 1.63(1.58-1.69) |
| Q5 (most deprived) | 2.11(1.95-2.29) | 2.06(1.99-2.12) |
| **Recent mental illness ^C^** |  |  |
| Unknown/No | 1.00 | 1.00 |
| Yes | 1.07(1.00-1.14) | 0.72(0.69-0.74) |
| **Recent Illicit drugs use ^C^** |  |  |
| Unknown/No | 1.00 | 1.00 |
| Yes | 25.04(23.7-26.44) | 6.17(6.00 -6.34) |
| **Recent Problematic alcohol use ^C^** |  |  |
| Unknown/No | 1.00 | 1.00 |
| Yes | 1.73(1.62-1.84) | 1.58(1.53-1.63) |

a Excluding unknown gender and health authority.; b Adjusted for health authority and year of diagnosis; c Recent is defined as 3 years prior to baseline - time of diagnosis for HCV positive, and date of last negative for HCV negative
